# SNPs in apolipoproteins contribute to sex-dependent differences in blood lipids before and after a high-fat dietary challenge in healthy U.S. adults

**DOI:** 10.1186/s40795-022-00592-x

**Published:** 2022-09-01

**Authors:** Yining E. Wang, Catherine P. Kirschke, Leslie R. Woodhouse, Ellen L. Bonnel, Charles B. Stephensen, Brian J. Bennett, John W. Newman, Nancy L. Keim, Liping Huang

**Affiliations:** 1 Integrative Genetics and Genomics, University of California at Davis, One Shields Ave, Davis, CA 95616 USA; 2 USDA/ARS/Western Human Nutrition Research Center, 430 West Health Sciences Drive, Davis, CA 95616 USA; 3 Department of Nutrition, University of California at Davis, One Shields Ave, Davis, CA 95616 USA

**Keywords:** Apolipoproteins, LDL receptor, Dietary challenge, Healthy adults, Lipid profile, LDL-C, HDL-C, Triglycerides, Cholesterol, NEFA

## Abstract

**Background:**

The effect of genetic polymorphisms on fasting blood lipid levels have been widely studied but the effects of these within the context of a high-fat meal challenge remain less characterized. The current study aimed to investigate the association of SNPs in lipoprotein-related genes with blood lipid profiles in healthy adults in the U.S.

**Methods:**

Subjects (*n* = 393) between 18–66 years of age with BMIs ranging from 18.5–45 kg/m^2^ were enrolled the cross-sectional Nutritional Phenotyping Study. Among them, 349 subjects (men: 48%; women: 52%) gave consent for genotyping. SNPs in *APOA5*, *APOB*, *APOC3*, *APOE*, and *LDLR* were assessed. The association between lipid markers and genotypes was tested separately for each SNP with analysis of variance (ANOVA), adjusted for sex, age, and BMI. We also examined two-factor interactions between SNPs and sex, age, or BMI.

**Results:**

Women carrying the C allele of rs3135506 in *APOA5* or men carrying the C allele of rs429358 in *APOE* had reduced HDL-cholesterol levels during fasting and postprandially. The C allele in *APOE* was also correlated to increased LDL-C levels. The TT genotype of rs2854116 in *APOC3* was associated with elevated total cholesterol. Additive effect of the risk alleles of *APOA5* and *APOE* or *APOC3* and *APOE* was detected. Nevertheless, the tested SNPs had little impact on the postprandial triglyceride responses to the high-fat challenge meal. We found no significant effects of SNPs in *APOB* (rs1042034) or *LDLR* (rs2228671) on triglycerides, cholesterol, or free fatty acid levels.

**Conclusions:**

In healthy adults, fasting and postprandial cholesterol levels are strongly correlated with the tested *APOA5*, *APOE*, and *APOC3* genotypes. Sex contributes to the genetic impact of the tested SNPs on lipid profiles.

**Trial registration:**

ClinicalTrials.gov, NCT02367287. Registered February 20, 2015, https://clinicaltrials.gov/ct2/show/NCT02367287.

**Supplementary Information:**

The online version contains supplementary material available at 10.1186/s40795-022-00592-x.

## Background

Cardiovascular diseases (CVDs) are one of the leading causes of death globally for over a decade and combined with Type 2 diabetes mellitus it has created a worldwide health epidemic [1]. The pathogenesis of CVDs is closely related to immune activation and inflammation, along with elevated circulating levels of triglycerides and cholesterol triggered by the consumption and accumulation of fat [2]. Lipoprotein particles are responsible for the transport of dietary and biosynthesized fats including cholesterol and triglycerides from the intestinal tract and liver to different sites of the body [3]. Apolipoproteins are recognized by a host of cell surface receptors in target tissues and thus play crucial roles in this lipid transport. Differences in lipoprotein particle densities, sizes, and apolipoprotein compositions help determine the tissue destination for particle uptake and usage [4].

Apolipoproteins are categorized based on their function, with the most well studied including the ApoAs, ApoBs, ApoCs, and ApoEs [5]. ApoAs with subtypes of ApoA1, ApoA2, ApoA4, and ApoA5 are the primary structural proteins of high-density lipoprotein particles (HDL). Importantly, ApoA5 is also associated with chylomicrons and very low-density lipoprotein particles (VLDL) to regulate triglyceride homeostasis [6–8]. Patients with ApoA5 deficiency are hypertriglyceridemic [9]. On the other hand, animals overexpressing the human ApoA5 decreases plasma triglyceride concentrations by 70% compared to the control [7, 8]. ApoB is the major structural protein for all lipoprotein particles, except HDL. ApoB100, the full-length form of ApoB, is a ligand for the LDL receptor (LDLR) which mediates the endocytosis of LDL from the circulation [10, 11]. Mutations in the *APOB* gene result in disorders of lipid metabolism, such as familial hypobetalipoproteinemia and familial ligand-defective ApoB100 [12]. In contrast, a high level of ApoB accompanied by high LDL particle concentrations is strongly associated with atherosclerosis and CVDs [13]. ApoC subtypes (ApoC1, ApoC2, and ApoC3) are associated with chylomicrons, VLDL, and HDL [14]. The ApoCs can be freely exchanged among these lipoprotein particles [5]. ApoC1 facilitates the esterification of free cholesterol into cholesterol esters in HDL [11], while ApoC2 is a co-factor for lipoprotein lipase (LPL), promoting triglyceride hydrolysis [15]. On the other hand, ApoC3 inhibits the ApoC2-mediated LPL activation [5, 11]. Importantly, ApoC3 can significantly reduce LDL clearance rate by inhibiting the receptor-mediated LDL endocytosis in the liver [16]. Thus, ApoC3 is considered as a powerful risk indicator for CVD risk and dyslipidemia [17]. ApoE is an essential apolipoprotein in cholesterol-rich lipoproteins, including chylomicron remnants, VLDL, and some HDL [18, 19]. This protein is also a ligand for LDLR-mediated hepatic LDL uptake. Three major allelic types of ApoE (ApoE2, ApoE3, and ApoE4) exist with different LDLR binding affinities [20]. Individuals carrying the homozygous *APOE4* allele have elevated plasma LDL and an increased risk of atherosclerosis and CVDs [18], while those carrying the homozygous *APOE2* allele have a high prevalence of type III hyperlipoproteinemia resulting in ectopic fat deposits in tissues and an elevated risk for atherosclerosis and diabetes [21]. LDLR is localized on the surface of hepatocytes where it binds to ApoB100 and ApoE directly affecting LDL clearance from the circulation [22, 23]. Mutations in the *LDLR* gene lead to familial hypercholesterolemia accompanied by significantly elevated LDL levels promoting atherosclerosis and CVDs [22].

Individual responses to dietary fat can differ in magnitudes and the kinetic behavior depends on both genetic, sex, and environmental factors [2]. Single-nucleotide polymorphisms (SNPs) in *APO* and *LDLR* genes, such as rs3135506 (*APOA5*), rs1042034 (*APOB*), rs2854116 (*APOC3*), rs429358 (*APOE*), and rs2228671 (LDLR) have been associated with dyslipidemia, including elevated cholesterol, triglycerides, or both along with increased LDL cholesterol (LDL-C) or decreased HDL cholesterol (HDL-C) levels in the circulation [14, 22, 24–26]. Unhealthy diets, such as those with high-fat contents, are thought to contribute to the degree of dyslipidemia leading to increased risk of developing atherosclerosis and CVDs [27]. In addition, studies in quantitative trait mapping and biological samples from an UK Biobank indicate genotype by sex interactions of lipid markers, apolipoproteins and lipoproteins have implications for the assessment of disease risk in men and women [28, 29]. Sexual dimorphism has been shown to be a contributor to the diversity of lipid profiles observed in men and women due to differences in fatty acid metabolic kinetics, postprandial modulation of adipose tissue lipolysis, rate of fat oxidation, fasting responses, plasma triglyceride kinetics, VLDL metabolism, and postprandial lipemia [30–34]. Moreover, genetic polymorphisms that manifest in a sex-dimorphic manner can potentially escalate the sexual disparities in lipid profiles [35].

While the impact of the above mentioned apolipoprotein gene SNPs on dyslipidemia have been studied extensively, the contributions of these SNPs in lipid clearance after a high-lipid meal challenge in healthy adults are less known, especially in healthy U.S. male and female adults differing in age, and/or BMI. Moreover, we do not understand how apolipoprotein gene SNPs may interact to influence postprandial lipid metabolism. The present study analyzed the contributions of *APOA5* (rs3135506), *APOB* (rs1042034), *APOC3* (rs2854116), *APOE* (rs429358), and *LDLR* (rs2228671) on clinical lipid markers before and after a high-fat liquid meal challenge in healthy U.S. individuals recruited for a cross-sectional nutritional phenotyping study conducted by the United States Department of Agriculture/Agriculture Research Service/Western Human Nutrition Research Center (WHNRC) in Davis, California. As sex, age, and BMI could be important effect modifiers on lipid metabolism during fasting and postprandial lipid removal from the circulation, the interplay between these variables and SNPs was also examined.

## Materials and methods

### Study subjects and dietary challenge

Healthy U.S. adults (*n* = 393) aged 18–66 years old with BMI ranges of 18.5 to 45.0 kg/m^2^ were enrolled for a cross-sectional Nutritional Phenotyping Study (*ClinicalTrials.gov*, ID: NCT02367287). For each sex group, 9 sampling bins were established to recruit subjects evenly among these bins. Three age bins, 18–33, 34–49, and 50–65 y were established, each with BMI (kg/m^2^) 18.5–24.9, 25.0–29.9, and 30–45.0. The original sampling plan had an upper limit of BMI at 39.9 kg/m^2^ [36]. But it was modified to increase the upper BMI to 45 kg/m^2^ to aid reaching recruitment goals. Pregnant or lactating women were excluded from the study. Other exclusion criteria included a known allergy to egg, recent surgeries or hospitalization (minor surgeries for the last 4 weeks or major surgeries/hospitalization for last 4 months), and antibiotic treatments for 4 weeks prior. Individuals who took daily medication for a diagnosed chronic disease at the time of the study were also excluded [36]. Additionally, subjects who took dietary supplements were instructed to discontinue the supplements for 3 days prior to the challenge meal test. Subjects were also instructed to only consume provided pre-test dinner and water the night before their tests. Details of recruitment and demographic characteristics of the overall population are contained in a report for this study [37].

The study included two visits to WHNRC scheduled within a period of 10–14 days. Visit 1 was the preliminary screening visit, which included an in-person informed consent procedure and a screening of vital signs to ensure the volunteers fell within expected ranges for the study. Visit 2 was the challenge meal test day. The night before the test day, the subject was provided a high carbohydrate meal (17% kcal from fat, 77% kcal from carbohydrate, and 7.5% kcal from protein) and asked to eat it by 19:00 h. The subject arrived fasted (12 h) the next morning and blood was collected before a high-fat liquid challenge meal (60% kcal from fat, 25% kcal from carbohydrates, and 15% kcal from protein) was given. Multiple blood draws were then conducted postprandially at 0.5-, 3- and 6-h [36]. Heights (m) and weights (kg) were recorded in Visit 1 and Visit 2. BMI (kg/m^2^) was then calculated from the averages of heights and the fasted weight from Visit 2. Waist circumference was measured using a non-elastic tape measure at the smallest horizontal circumference between the ribs and iliac crest [36].

### SNP selection

A single SNP in each of 4 apolipoprotein genes, *APOA5*, *APOC3*, *APOB*, and *APOE*, and the *LDLR* gene were chosen in this study. We chose these SNPs based on their strong correlations with lipid metabolism-related diseases, such as stroke or metabolic syndrome revealed in multiple genome-wide association and meta-analysis studies [26, 38–45], and their relative high risk allele carrier frequencies, which would increase detection power for this particular study with relatively small participant numbers. Table 1 lists the SNP IDs for the chosen SNPs and their corresponding TaqMan assay IDs, allele frequencies in populations, nucleotide changes, codon changes, physical positions in the human genome, and the associated metabolic diseases. Four of the chosen SNPs in *APOA5*, *APOB*, *APOE*, and *LDLR* lead to missense amino acid changes in the respective protein. The SNP in *APOC3* is located upstream of the *APOC3* coding sequence with a potential role in regulation of *APOC3* gene expression [44, 46].

**Table 1:** SNPs used in this study

Gene	SNP ID	TaqMan assay ID	Nucleotide Change	Frequency ^*a*^	Codon change	Genome position (GRCh38.p12)	Associated diseases with null mutations
*APOA5*	rs3135506	C_25638153_10	C > G	C = 0.056 (279/5008)	S[TCG] > W[TGG]	chr11:116,791,691	Familial hypertriglyceridemia
*APOB*	rs1042034	C_7615376_20	G > A	G = 0.370 (1855/5008)	S[AGT] > N[AAT]	chr2:21,002,409	Familial hypercholesterolemia II
*APOC3*	rs2854116	C_12081482_20	C > T	T = 0.452 (2262/5008)	n.a. ^*b*^	chr11:116,829,453	Hypertriglyceridemia, Nonalcoholic fatty liver disease
*APOE*	rs429358	C_3084793_20	C > T	C = 0.151 (754/5008)	R[CGC] > C[TGC]	chr19:44,908,684	Hyperlipoproteinemia type III
*LDLR*	rs2228671	C_27208873_10	C > T	T = 0.057 (285/5008)	C[TGC] > C[TGT]	chr19:11,100,236	Familial hypercholesterolemia I

^a^ Based on the data from the 1000 Genomes Project (https://www.internationalgenome.org/)

^b^ Upstream Transcript Variant

### Genomic DNA purification and quantification

Eight milliliters of whole blood were collected in a PAXgene Blood DNA Tube (Qiagen, Germantown, MD) from study subjects at 0.5 h after the challenge meal was given. Collected blood was gently inverted in the PAXgene blood DNA tube and immediately stored at -80 °C until use. Genomic DNA was then purified from the whole blood using a PAXgene Blood DNA Kit according to the manufacturer’s instructions (Qiagen). The concentrations of DNA were measured using a NanoPhotometer™ P300 (Implen, Westlake Village, CA, USA). All DNA samples had a A260/A280 ratio greater than 1.6, indicating the purity of nucleic acids was suitable for genotyping. DNA was then diluted to 25 ng/µL for TaqMan SNP genotyping with sterilized double distilled water for subsequent TaqMan SNP genotyping assays.

### SNP genotyping

TaqMan SNP probe sets for rs3135506 (Assay ID: C_25638153_10, *APOA5*), rs1042034 (Assay ID: C_7615376_20, *APOB*), rs2854116 (Assay ID: C_12081482_20, *APOC3*), rs429358 (Assay ID: C_3084793_20, *APOE*), rs2228671 (Assay ID: C_27208873_20, *LDLR*) were purchased from ThermoFisher Scientific (Carlsbad, CA, USA). TaqMan genotyping reactions were performed using a TaqMan SNP assay-based PCR (ThermoFisher Scientific) in an Applied Biosystems™ QuantStudio™ 7 Flex Real-Time PCR System according to the manufacturer’s instructions. Fifty ng of genomic DNA was used for each PCR reaction. Allelic discrimination assays were performed using QuantStudio™ Real-Time PCR software (ThermoFisher Scientific). All ambiguous genotypes were repeated in independent PCR reactions.

### Clinical measures

Blood was collected and serum or plasma was obtained by centrifugation at 1300 × g at 4 °C for 10 min. Lipid-related markers of cardiovascular diseases, including triglycerides (TG), total cholesterol (TC), HDL-C, LDL-C, and non-esterified free fatty acids (NEFA) were measured using a Cobas Integra 400/800 kit (Roche), a Cobas CHOL2 kit (Roche), a Cobas HDL-C plus 3^rd^ generation kit (Roche), a Cobas LDLC3 kit (Roche), and a Wako HR Series NEFA-HR (2) kit (Wako), respectively. All assays were completed on an auto-analyzer, Integra 400 + instrument (Roche).

### Statistical analysis

Allele frequency was determined by direct counting. Differences in general characteristics between genotype groups were assessed for significance using Student’s *t*-test. Outcome variables were assessed for conformance to the normal distribution via Box-Cox power transformations and transformed if needed; triglycerides were transformed using natural logarithm, and total cholesterol, HDL-C, LDL-C, and NEFA did not require transformation. The association between lipid parameters and genotypes was tested separately for each SNP with analysis of variance (ANOVA), adjusted for sex, age, and BMI. Two-factor interactions between SNP and sex, age, or BMI were also examined. The general characteristics were presented as mean ± S.E. *P* < 0.05 was considered significant.

## Results

### General results

**Fig. 1:**
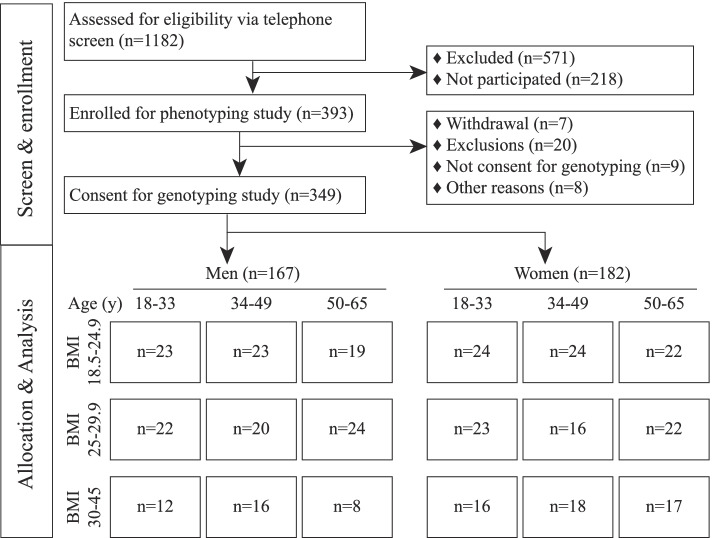
Study flow diagram documenting participant enrollment, allocation, and analysis. Sex, age in years (y), and BMI in kg/m^2^ are shown for each allocation bin

The objective of this study was to understand potential genetic contributions of apolipoprotein (*APOA5*, *APOB*, *APOC3*, *APOE*) and *LDLR* genes to clinical lipid measures, including total cholesterol (TC), HDL-C, LDL-C, triglycerides (TG), and non-esterified free fatty acids (NEFA), during fasting and after a high-fat liquid challenge meal. Figure 1 provides a complete diagram of participant enrollment, allocation, and analysis. A total of 393 subjects were enrolled and completed the nutritional phenotyping study, with genomic DNA available from 349 subjects (88.8% of the total enrolled subjects). The analysis cohort consisted of 167 men (48%) and 182 women (52%). Among the men, 57 (34.1%), 59 (35.3%), and 51 (30.5%) were in the age groups of 18–33, 34–49, and 50–65 years old, respectively. Among the women, 63 (34.1%), 58 (31.9%), and 61 (33.5%) were in the age groups of 18–33, 34–49, and 50–65 years old, respectively. A total of 135 (65 men and 70 women), 127 (66 men and 61 women) and 87 (36 men and 51 women) subjects were in the BMI (kg/m^2^) groups of 18.5–24.9 (normal weight), 34–29.9 (overweight), and 30–45 (obese), respectively.

**Fig. 2:**
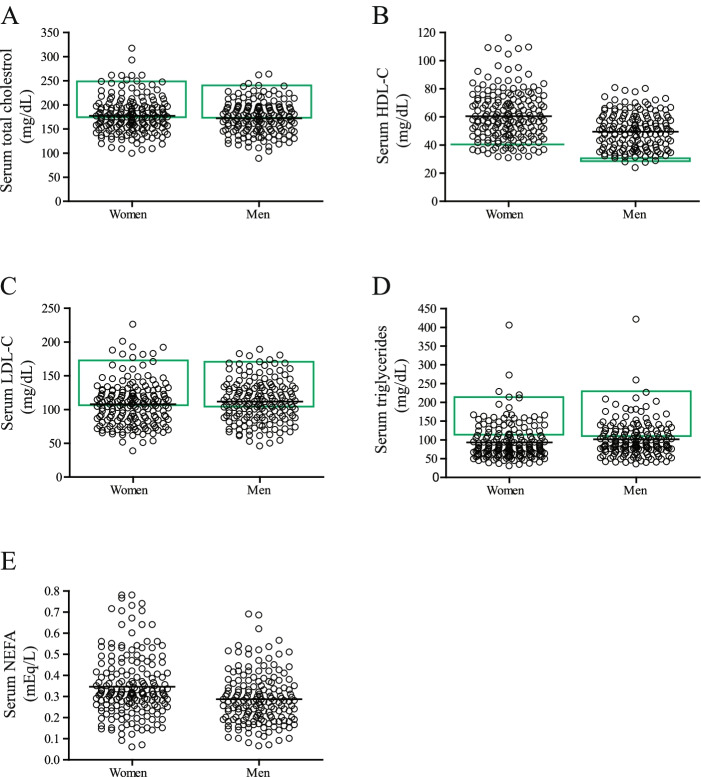
Distributions of fasting lipid measures in subjects. Each open circle represents a subject and the black line is the mean of the dataset. The green line or box represents the reference values for 18.5- to 65-year-old categories obtained from the Lipid Research Clinic (LRC) Program Population Studies [47]. The 90^th^ percentile (triglycerides), 75^th^ percentile (cholestrol and LDL-C), and 10^th^ percentile (HDL-C) of the LRC datasets in different age groups were used for determining the reference values. Lower (triglycerides, total cholestrol, and LDL-C) or higher (HDL-C) concentrations than the reference values are desired for reducing the risk of coronary artery disease

The general characteristics, including age, height, weight, BMI, and waist circumference are shown for the genotyped participants in Table 2. Higher weight, height, and waist circumference were present in men than women (*P* < 0.01). Fasting blood lipid profiles, including TC, HDL-C, LDL-C, TG, and NEFA from 349 subjects are presented in Fig. 2. Most of subjects had fasting lipid values within the desirable ranges for healthy U.S. individuals in the respective age group based on the lipid reference values obtained from the Lipid Research Clinic (LRC) Program Population Studies [47]. Reference values for TC are 170—235 mg/dL for men and 175—250 mg/dL for women; LDL-C are 105—165 mg/dL for men and 110—170 mg/dL for women; HDL-C are 30—35 mg/dL for men and 40 mg/dL for women, and triglycerides are 120—210 mg/dL for men and 115—205 mg/dL for women. The reference values for fasting plasma NEFA concentrations in men and women have not been established. In this study, we observed that the average values for fasting plasma NEFA concentrations (mEq/L) were 0.29 ± 0.01 for men and 0.35 ± 0.01 for women. Women had approximately 20% higher fasting NEFA concentrations than men (*P* < 0.01). The NEFA levels were also positively associated with BMI (0.29 ± 0.01 mEq/L in the BMI 18.5–24.9 group; 0.31 ± 0.01 mEq/L in the 25.0–29.9 group, and 0.39 ± 0.01 mEq/L in the 30.0–45.0 group; *P* < 0.0001). Age-dependent increases in NEFA levels approached significance overall (0.30 ± 0.01 mEq/L in the 18.5–33 y group; 0.33 ± 0.01 mEq/L in the 34–49 y group; and 0.34 ± 0.01 mEq/L in the 50–65 y group; *P* = 0.054). As expected, women had higher HDL-C than men (*P* < 0.01).

**Table 2:** General characteristics of participants

	Men	Women
N	167 (48%)	182 (52%)
Age (y)	39.7 ± 14.0 (38.0; 18.0–65.0)	40.6 ± 13.7 (41.0; 19.0–65.0)
Height (cm) ^*a*^	177.9 ± 7.6 (177.4; 161.8–201.7)	163.6 ± 6.9 (163.9; 146.3–180.6)
Weight (kg) ^*a*^	86.2 ± 17.8 (84.2; 50.3–175.2)	74.6 ± 16.2 (71.9; 44.9–121.2)
Body mass index (kg/m^2^)	27.1 ± 4.7 (26.2; 18.2–43.9)	27.8 ± 5.3 (27.1; 18.0–43.3)
Waist circumference (cm) ^*a*^	88.7 ± 12.8 (86.0; 63.3–138.4)	82.6 ± 12.2 (80.4; 60.8–119.7)

Data are shown as number (percentage) and metric data as mean ± SD (median; range)

^a^ statistically significant between men and women (*P* < 0.05)

### SNP carrier frequency

Among the genotyped participants, 6.88% of the total had a CC or CG genotype of rs3135506 (*APOA5*) with the dominant C genotype being the risk allele for its association with hypertriglyceridemia and cardiovascular diseases [48]. In contrast, 26.16% of the subjects had a risk GG or GA genotype of rs1042034 (*APOB*) for its correlation to hyperlipidemia and ischemic stroke [49, 50]. In addition, 42.22% of the subjects carried the dominant C risk allele of rs2854116 (*APOC3*) for either hypertriglyceridemia or nonalcoholic fatty liver disease [51, 52]. Moreover, 14.33% of the subjects had a CC or CT genotype of rs429358 (*APOE*) with the dominant C genotype being the risk allele for coronary heart disease and Alzheimer's disease [53, 54]. Lastly, 9.34% of the subjects had a T genotype of rs2228671 (*LDLR*) with the dominant T genotype being the risk allele for hypercholesterolemia [55]. The comparison of allelic frequencies of the tested SNPs with the ones in the database of the 1000 Genomes Project (https://www.genome.gov/27528684/1000-genomes-project) is presented in Supplemental Table 1. The allele frequencies of the SNPs revealed in the current study were in agreement with the ones derived from the global population in the database. In addition, these observed SNP genotypes were evenly distributed across all age/BMI/sex groups (Supplemental Table 2).

### Association of SNPs in *APOA5*, *APOB*, *APOC3*, *APOE*, and *LDLR* with fasting TC, HDL-C, LDL-C, TG, and NEFA levels

Most of the tested SNPs (4 out 5) were expected to have a dominant effect on protein function due to missense amino acid changes. Thus, a dominant nucleotide model was adopted for each SNP for analyzing the association of the SNP with clinical lipid measures, such as CC + CG vs GG for rs3135506 (*APOA5*); GG + AG vs AA for rs1042034 (*APOB*), CC + CT vs TT for rs2854116 (*APOC3*) as well as rs429358 (*APOE*), and CT + TT vs CC for rs2228671 (*LDLR*). As shown in Table 3, the adjusted mean corrected for sex, age, and BMI for fasting LDL-C concentrations were higher by 8% (8.8 mg/dL; *P* < 0.05) in the subjects carrying the CC or CT genotype of rs429358 (*APOE*). On the other hand, the adjusted mean for fasting HDL-C were reduced in these subjects by 7% (3.7 mg/dL; *P* < 0.05). Moreover, a significant association was detected for the SNP in *APOC3* for fasting TC levels. Subjects carrying the CC or CT genotype of rs2854116 (*APOC3*) had higher TC levels by 5% (8.0 mg/L; *P* < 0.05) than those carrying the TT genotype. Additionally, fasting LDL-C levels were elevated in these subjects with high TC levels; but it did not reach statistical significance (*P* = 0.06). Interestingly, no correlation of the SNPs in *APOA5*, *APOB* and *LDLR* could be established with TG, TC, HDL-C, LDL-C, and NEFA levels in the fasting state although the adjusted mean for fasting HDL-C levels were lower by 7% (4.0 mg/dL; *P* = 0.08) in individuals carrying the dominant C risk allele of the *APOA5* SNP. Moreover, no significant association of the tested SNPs in *APOE* and *APOC3* with fasting TG and NEFA levels could be found (Table 3).

**Table 3:** Fasting blood lipid concentrations based on SNPs in the examined genes

Gene & genotype (n)	TG (mg/dL)	Total cholesterol (mg/dL)	HDL-C (mg/dL)	LDL-C (mg/dL)	NEFA (mEq/L)
*APOA5*					
CC (1) + CG (46)	90.69 ± 5.20	179.3 ± 4.67	52.04 ± 1.88	116.7 ± 4.23	0.31 ± 0.02
GG (302)	87.72 ± 1.92	174.2 ± 1.81	55.78 ± 0.96	108.7 ± 1.64	0.32 ± 0.01
% change of the C allele	3.27	2.84	-7.19	6.86	-3.23
*APOB*					
GG (27) + AG (127)	88.57 ± 2.80	174.7 ± 2.57	56.43 ± 1.10	108.9 ± 2.33	0.32 ± 0.01
AA (192)	87.74 ± 2.48	175.0 ± 2.30	54.39 ± 0.98	110.4 ± 2.08	0.32 ± 0.01
% change of the G allele	0.95	-0.17	3.62	-1.38	0
*APOC3*					
CC (63) + CT (167)	87.74 ± 2.25	177.5 ± 2.07	55.95 ± 0.90	111.8 ± 1.89	0.33 ± 0.01
TT (117)	88.30 ± 3.19	169.5 ± 2.93	54.01 ± 1.27	105.7 ± 2.67	0.30 ± 0.01
% change of the C allele	-0.64	4.51 ^*^	3.47	5.46	9.09
*APOE*					
CC (4) + CT (92)	93.08 ± 3.74	179.1 ± 3.28	52.62 ± 1.41	116.2 ± 2.96	0.33 ± 0.01
TT (253)	86.30 ± 2.11	173.2 ± 1.99	56.29 ± 0.85	107.4 ± 1.80	0.31 ± 0.01
% change of the C allele	7.28	3.29	-6.97 ^*^	7.57 ^*^	6.06
*LDLR*					
CT (59) + TT (3)	88.98 ± 4.43	178.8 ± 4.05	57.73 ± 1.74	110.8 ± 3.69	0.32 ± 0.02
CC (286)	87.94 ± 2.02	174.0 ± 1.87	54.74 ± 0.80	109.5 ± 1.70	0.32 ± 0.01
% change of the T allele	1.17	2.68	5.18	1.18	0

Data are presented as mean ± S.E

^*^ *P* < 0.05. Triglyceride (TG) values were transformed to the natural logarithm scale for analysis. Other analyses were conducted without transformation. Data were adjusted for sex, age, and BMI. The percent (%) change of each lipid level due to carrying the risk allele of each SNP was calculated by dividing the mean value of the difference between two genotypes of the SNP by the mean value of the risk allele and multiply the answer by 100

Since the postprandial TG response is well-known to be increased in the circulation after a high-fat intake [56] and genotypes of SNPs in *APO* and *LDLR* genes may have an impact on the postprandial TG metabolism, we therefore investigated the postprandial TG response in subjects carrying different genotypes of the tested SNPs separated by sex. We observed that serum TG levels increased slightly at 30 min post the dietary challenge and reached to the peak at 3 h postprandially (Supplemental Fig. 1). We also noticed that men had greater increase in the TG levels at 3- and/or 6-h after the challenge than women with the same genotypes of the SNPs (Supplemental Fig. 1A-E). However, no significance was detected for the interaction of SNP genotypes with postprandial TG changes in men or women in this study.

Genotype-sex interactions of apolipoproteins with HDL-C levels have been reported [57]. We, therefore, investigated sex-specific changes in HDL-C levels that were associated with the tested genotypes of the apolipoprotein genes and LDLR. As shown in Table 4, women had significantly higher fasting HDL-C levels than men regardless of their tested genotypes (*P* < 0.01) except for the CC or CG genotype of rs3135506 (*APOA5*; *P* > 0.05; Table 4). Women carrying the CC or CG risk genotype of rs3135506 had ~ 17% (9 mg/dL) lower HDL-C than those with the GG genotype (*P* < 0.05). However, this difference was not detected in men (*P* > 0.05). Specifically, women carrying the C dominant risk allele of rs3135506 had HDL-C levels similar to men. Moreover, men carrying the C allele of rs429358 (*APOE*) had significantly lower HDL-C levels (~ 12%; 5 mg/dL) than those with the TT genotype (Table 4) whereas it was not noted in women. Taken together, sex appeared to contribute to the association of genotypes of rs3135506 (*APOA5*) and rs429358 (*APOE*) with HDL-C levels (*P* < 0.05 after adjustment for age and BMI).

**Table 4:** Fasting serum HDL-C based on SNPs and sexes in the examined genes

Men		Women	
Gene & genotype (n)	HDL-C (mg/dL)	Gene & genotype (n)	HDL-C (mg/dL)
*APOA5*		*APOA5*	
CC (0) + CG (25)	50.57 ± 2.72	CC (1) + CG (21)	52.88 ± 2.87
GG (142)	49.00 ± 1.13	GG (160)	61.91 ± 1.06 ^*a*^
% change of the C allele	3.10%	% Change of the C allele	-17.08^*c*^
*APOB*		*APOB*	
GG (12) + AG (60)	49.16 ± 1.60	GG (15) + AG (67)	63.28 ± 1.50 ^*a*^
AA (93)	49.51 ± 1.40	AA (99)	58.60 ± 1.38 ^*a*^
% change of the G allele	-0.71	% Change of the G allele	7.40
*APOC3*		*APOC3*	
CC (28) + CT (80)	50.53 ± 1.31	CC (35) + CT (87)	61.10 ± 1.23 ^*a*^
TT (58)	46.94 ± 1.79	TT (59)	60.24 ± 1.78 ^*a*^
% change of the C allele	7.10	% Change of the C allele	1.41
*APOE*		*APOE*	
CC (2) + CT (38)	45.22 ± 2.16	CC (2) + CT (54)	59.45 ± 1.82 ^*a*^
TT (127)	50.50 ± 1.21	TT (126)	61.43 ± 1.21 ^*a*^
% change of the C allele	-11.68 ^*b*^	% Change of the C allele	-3.33
LDLR		LDLR	
CT (29) + TT (2)	50.61 ± 2.45	CT (30) + TT (1)	64.46 ± 2.45 ^*a*^
CC (135)	48.89 ± 1.17	CC (151)	60.06 ± 1.11^*a*^
% change of the T allele	3.40	% Change of the T allele	6.83

Data were adjusted for age and BMI in each sex group and are presented as mean ± S.E

^a^ *P* < 0.01 between men and women of the indicated genotype

^b^ *P* < 0.05

^c^ *P* < 0.01 between genotypes of the indicated gene. The percent (%) change of HDL-C levels due to carrying the risk allele of each SNP was calculated by dividing the mean value of the difference between two genotypes of the SNP by the mean value of the risk allele and multiply the answer by 100

### The effects of SNPs in *APOA5* and *APOE* on postprandial HDL-C levels after lipid challenge

**Fig. 3:**
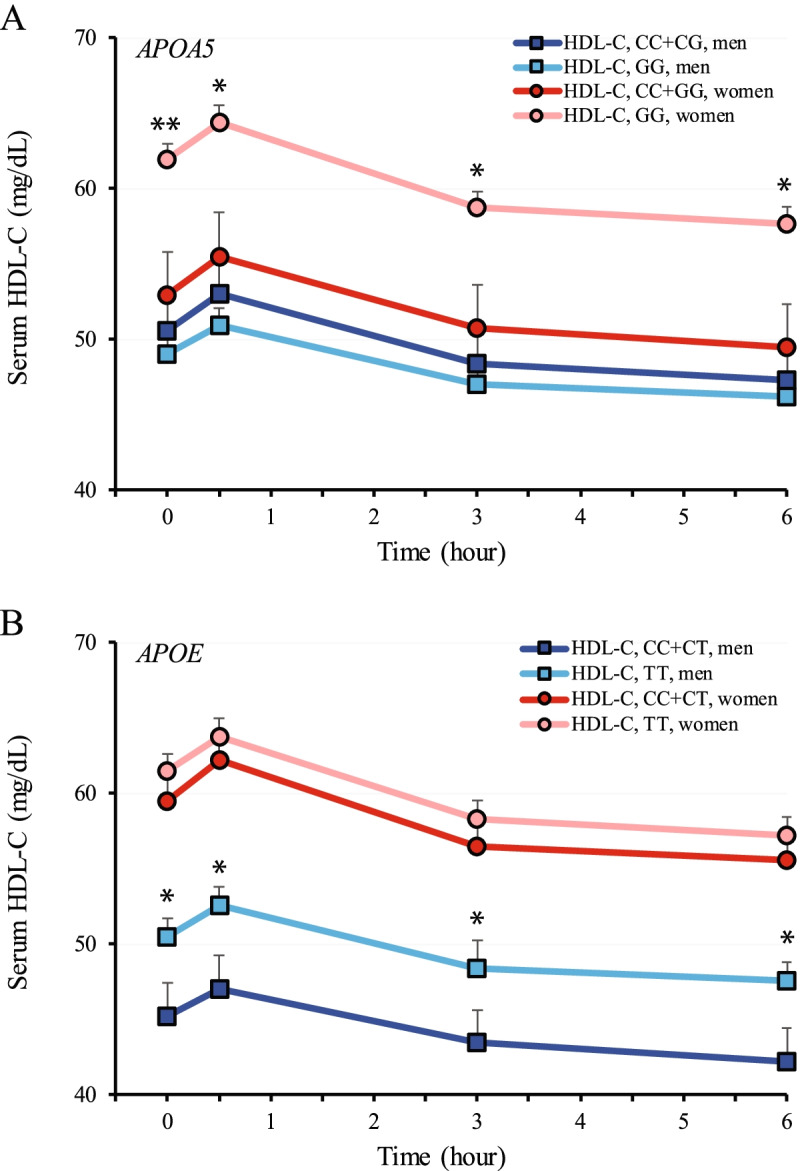
Effects of *APOA5* rs23135506 and *APOE* rs429358 on serum HDL-C levels after lipid challenge. A. *APOA5*. Subjects were divided into two genotypic groups separated by sex (*n* = 25 and = 22 for the CC + CG genotype in men and women, respectively; *n* = 142 and = 160 for the GG genotype in men and women, respectively). B. *APOE*. Subjects were divided into two genotypic groups separated by sex (*n* = 40 and = 56 for the CC + CT genotype in men and women, respectively; *n* = 127 and = 126 for the TT genotype in men and women, respectively). All data were adjusted for age and BMI and are presented as mean ± S.E. *, *P* < 0.05 and **, *P* < 0.01 between genotypic groups within the same sex

To further analyze the association of SNPs in *APOA5* and *APOE* with the metabolic pattern of HDL-C, an ANOVA was performed to draw associations between the SNPs and HDL-C levels at four different time points in men and women, which were 0- (before lipid challenge); 0.5-, 3-, and 6-h after lipid challenge (Fig. 3). Our results demonstrated that women carrying the dominant C genotype (risk allele) of the *APOA5* SNP had significantly decreased HDL-C levels across all time-points by ~ 17% (9 mg/dL) compared to those with the GG genotype. Moreover, the risk *APOE* C allele male carriers had lower levels of HDL-C than the non-carriers by ~ 12% (5 mg/dL) before and after lipid challenge. Taken together, these results demonstrated that the risk alleles of *APOA5* (rs3135506) and *APOE* (rs429358) SNPs negatively affected cholesterol concentrations in HDL particles in a sex-dependent manner on the genotype by sex interaction after adjustment for age and BMI (*P* < 0.05). However, the postprandial clearance patterns of HDL-C after lipid challenge was not affected by the genotypes of the SNPs in *APOA5* and *APOE* in both men and women, suggesting a baseline effect of these SNPs on HDL-C levels.

### The effects of SNPs in *APOC3* and *APOE* on postprandial TC and LDL-C levels after lipid challenge

**Fig. 4:**
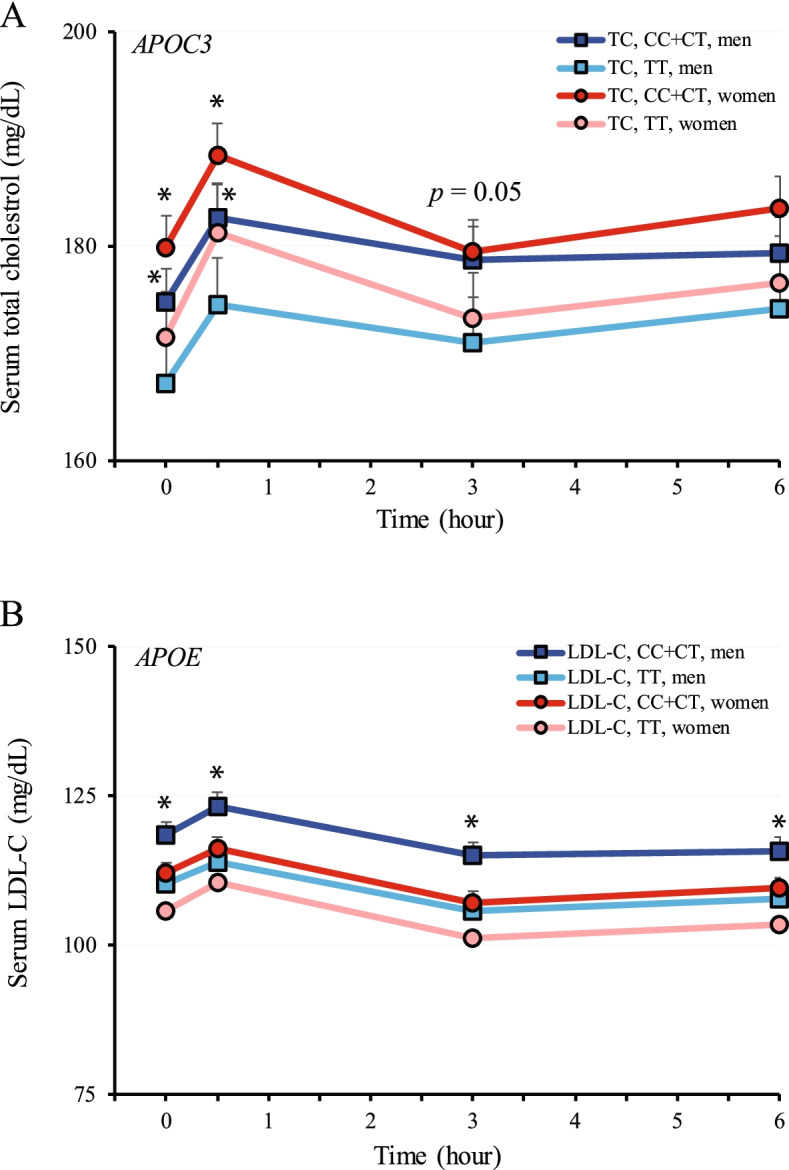
Effects of *APOC3* rs2854116 and *APOE* rs429358 on blood TC and LDL-C levels after lipid challenge. A. *APOC3*. Subjects were grouped into two genotypic groups separated by sex (*n* = 108 and = 122 for the CC + CT genotype in men and women, respectively; *n* = 58 and = 59 for the TT genotype in men and women, respectively). B. *APOE*. Subjects were grouped into two genotypic groups and separated by sex (*n *= 40 and = 56 for the CC + CT genotype in men and women, respectively; *n* = 127 and = 126 for the TT genotype in men and women, respectively). All data were adjusted for age and BMI and are presented as mean ± S.E. *, *P* < 0.05 between two genotypic groups within the same sex. *P* = 0.05 between two genotypes within the same sex. TC = total cholestrol

In the current study, interactions of the SNPs in *APOC3* and *APOE* with TC or LDL-C concentrations were revealed in the fasting state (Table 3). We further assessed postprandial TC or LDL-C levels in different genotypic and sex groups following lipid challenge. As shown in Fig. 4A, subjects of both sexes with the dominant *APOC3* C genotype (risk allele) had significantly higher TC concentrations (~ 4%; 8 mg/dL after adjustment for age and BMI) than those with the TT genotype at 0- and 0.5-h post lipid challenge. TC levels tended to remain at higher levels in subjects carrying the risk allele at 3- and 6-h post challenge. However, it did not reach statistical significance for both time points (*P* = 0.05 for the 3-h time point and *P* = 0.08 for the 6-h time point). Moreover, we found that the *APOE* SNP-mediated difference in fasting LDL-C levels (Table 3) was mainly contributed by men (*P* < 0.05 on the genotype by sex interaction after adjustment for age and BMI). As shown in Fig. 4B, men carrying the dominant C allele had ~ 7% (8 mg/dL) higher LDL-C levels than those with the TT allele (*P* < 0.05) and this difference remained postprandially. A similar trend was observed in women before and after lipid challenge. However, no statistical significance was detected (*P* > 0.05). Again, it was noted that the postprandial clearance patterns of TC or LDL-C was not influenced by either *APOC3* or *APOE* SNP after lipid challenge (Fig. 4A and B).

### Additive effects of the SNPs in *APOA5*/*APOE* and *APOC3*/*APOE* on lipid markers

The risk genotypes of SNPs in *APOA5* (*P* = 0.08) and *AOPE* (*P* < 0.05) displayed negative correlations with HDL-C levels at fasting (Table 3). Therefore, we examined whether the carriers for both *APOA5* and *APOE* risk alleles showed a difference in serum lipid levels, i.e., a potential additive effect of the risk alleles on the levels of TC, HDL-C, LDL-C, TG, and NEFA before and after lipid challenge. As shown in Table 5, 18 of the 349 genotyped subjects were carriers for both risk alleles of *APOA5* and *APOE* SNPs, while 224 of them were non-carriers. We observed an additive effect of risk alleles of *APOA5* and *APOE* for HDL-C (~ 10–12% or ~ 6 mg/dL) throughout the 6-h course of the lipid challenge. However, it was not statistically significant after the data were adjusted for age, sex, and BMI (*P* > 0.05), indicating that the difference was largely driven by other independent variables rather than SNP genotypes (Table 5). Importantly, fasting LDL-C was significantly increased by ~ 12% (15 mg/dL) in subjects carrying both risk alleles of *APOA5* and *APOE* compared to the non-carriers (*P* < 0.05). This difference was maintained postprandially (*P* < 0.05), suggesting the effect at the baseline might be the causal effect on LDL-C levels. Nevertheless, other lipid measures, such as TC, TG, and NEFA, were not significantly affected by the combination of the risk alleles of *APOA5* and *APOE* (Table 5). Lastly, subjects carrying the double risk alleles of *APOA5* and *APOE* had a similar postprandial clearance patterns of HDL-C and LDL-C that were similar to these carrying the non-risk alleles of *APOA5* and *APOE* (Table 5).

**Table 5:** Lipid concentrations after dietary challenge in subjects carrying both risk alleles of *APOA5* and *APOE*

Gene & genotype (n)	Time (h) after dietary challenge			
	0	0.5	3	6
*APOA5/APOE*, GG/TT (224) ^*a*^				
Triglycerides (mg/dL)	87.05 ± 2.3	97.30 ± 2.6	178.5 ± 5.5	148.3 ± 4.7
Total cholesterol (mg/dL)	172.8 ± 2.11	180.8 ± 2.24	174.3 ± 2.16	176.9 ± 2.20
HDL-C (mg/dL)	56.07 ± 0.88	58.20 ± 0.91	53.35 ± 0.88	52.48 ± 0.88
LDL-C (mg/dL)	107.1 ± 1.90	111.2 ± 1.99	102.4 ± 1.84	104.8 ± 1.90
NEFA (mEq/L)	0.32 ± 0.01	0.23 ± 0.01	0.26 ± 0.01	0.56 ± 0.01
*APOA5/APOE*, CC + CG/CC + CT (18) ^*b*^				
Triglycerides (mg/dL)	93.09 ± 9.0	101.8 ± 9.9	199.7 ± 22.8	170.6 ± 19.9
% change of the risk alleles	6.49	4.42	10.62	13.07
Total cholesterol (mg/dL)	182.2 ± 7.85	191.7 ± 8.30	185.9 ± 8.00	189.1 ± 8.09
% change of the risk alleles	5.16	5.69	6.24	6.45
HDL-C (mg/dL)	49.99 ± 3.28	52.96 ± 3.35	47.90 ± 3.25	47.30 ± 3.23
% change of the risk alleles	-12.16	-9.89	-11.38	-10.95
LDL-C (mg/dL)	122.0 ± 7.07	127.0 ± 7.36	117.8 ± 6.83	119.3 ± 6.99
% change of the risk alleles	12.21 ^*^	12.44 ^*^	13.07 ^*^	12.15 ^*^
NEFA (mEq/L)	0.30 ± 0.03	0.21 ± 0.03	0.27 ± 0.03	0.52 ± 0.04
% change of the risk alleles	-6.67	-9.52	3.70	-7.69

Data were adjusted for sex, age, and BMI and are presented as mean ± S.E

^a^ subjects carrying both non-risk alleles of *APOA5* and *APOE*

^b^ subjects carrying both risk alleles of *APOA5* and *APOE*

^*^P < 0.05. The percent (%) change of each lipid level due to carrying the combination of the risk alleles of *APOA5* and *APOE* was calculated by dividing the mean value of the difference between CC + CG/CC + CT and GG/TT genotypes of *APOA5*/*APOE* by the mean value of CC + CG/CC + CT genotype and multiply the answer by 100

Additive effects of the risk alleles of *APOC3* and *APOE* SNPs on lipid markers were also evaluated in both fasting and postprandial states. As shown in Table 6, carriers of the double risk alleles (CC or CT for both *APOC3* and *APOE*; *n* = 21) had lower NEFA concentrations 6-h after lipid challenge (decreased ~ 24% or 0.11 mEq/L; *P* < 0.01 after adjustment for age, sex, and BMI) than the non-carriers (*n* = 130). Nonetheless, the presence of the double risk alleles of *APOC3* and *APOE* SNPs had limited impact on other tested lipid markers before and after lipid challenge.

**Table 6:** Lipid concentrations after dietary challenge in subjects carrying both risk alleles of *APOC3* and *APOE*

Gene & genotype (n)	Time (h) after dietary challenge			
	0	0.5	3	6
*APOC3/APOE*, TT/TT (130) ^*a*^				
Triglycerides (mg/dL)	87.14 ± 2.94	97.17 ± 3.45	176.2 ± 7.02	148.6 ± 6.53
Total cholesterol (mg/dL)	178.0 ± 2.98	186.0 ± 3.15	179.1 ± 3.09	181.6 ± 3.13
HDL-C (mg/dL)	55.78 ± 1.30	58.00 ± 1.36	52.90 ± 1.29	52.35 ± 1.30
LDL-C (mg/dL)	112.6 ± 2.80	116.7 ± 2.91	107.8 ± 2.73	109.6 ± 2.80
NEFA (mEq/L)	0.33 ± 0.01	0.24 ± 0.01	0.25 ± 0.01	0.56 ± 0.01
*APOC3/APOE*, CC + CT/CC + CT (21) ^*b*^				
Triglycerides (mg/dL)	86.48 ± 7.53	96.38 ± 9.08	172.3 ± 17.66	140.5 ± 15.72
% change of the risk alleles	-0.76	-0.82	-2.26	-5.77
Total cholesterol (mg/dL)	184.1 ± 7.70	193.7 ± 8.35	186.6 ± 7.93	189.3 ± 8.02
% change of the risk alleles	3.31	3.98	4.02	4.07
HDL-C (mg/dL)	55.00 ± 3.36	57.59 ± 3.61	53.36 ± 3.12	52.22 ± 3.33
% change of the risk alleles	-1.42	-0.71	0.86	-0.25
LDL-C (mg/dL)	121.5 ± 7.23	127.1 ± 7.72	117.7 ± 7.01	119.7 ± 7.16
% change of the risk alleles	7.33	8.18	8.41	8.44
NEFA (mEq/L)	0.29 ± 0.03	0.23 ± 0.03	0.21 ± 0.02	0.45 ± 0.03
% change of the risk alleles	-13.79	-1.72	-15.96	-24.28 ^**^

Data were adjusted to sex, age, and BMI and are presented as mean ± S.E

^a^ subjects carrying both non-risk alleles of *APOC3* and *APOE*

^b^ subjects carrying both risk alleles of *APOC3* and *APOE*

^**^ *P* < 0.01. The percent (%) change of each lipid level due to carrying the combination of the risk alleles of *APOC3* and *APOE* was calculated by dividing the mean value of the difference between CC + CT/CC + CT and TT/TT genotypes of *APOC3*/*APOE* by the mean value of CC + CT/CC + CT genotype and multiply the answer by 100

## Discussion

In this study, we aimed to draw an association between lipid-metabolic parameters and SNPs in apolipoprotein and their receptor genes and to assess their impact on postprandial lipid patterns following a liquid high-fat meal challenge. Of all the clinical lipid markers studied, total cholesterol, HDL-C, and LDL-C were the ones with significant associations with the tested SNPs in *APOA5*, *APOE*, and *APOC3*.

We showed that the risk allele of rs3135506 in *APOA5* was significantly associated with serum HDL-C levels in a sex-dependent manner. Specifically, the dominant risk C allele of the SNP was associated with lower HDL-C levels in women (Fig. 3). It is known that HDL-C levels differ between men and women. In general, women have higher HDL-C than men [58]. Our results demonstrate that this sex difference is diminished in women carrying the risk allele of rs3135506. Clinically, HDL-C is known as “the good cholesterol” as studies showed its association with a lower risk of coronary heart disease [59–62]. It is commonly recognized that HDL-C exerts its protective effects towards arteries and the heart by transporting cholesterol away from extrahepatic tissues, particularly steroidogenic tissues. By doing so, it removes cholesterol from the arteries, delivering them to the liver [59–62]. The current study is the first report to show an association of rs3135506 with HDL-C levels in healthy women before and after a meal challenge. Our study suggests that the risk allele of rs3135506 could act as an independent cardiovascular risk factor for women who carry the risk allele.

ApoA5 is an activator of lipoprotein lipase [8] and null mutations in the human *APOA5* gene leads to high blood TG levels [9]. Therefore, its role in TG metabolism and the therapeutic modification of TG levels have been major research topics. Surprisingly, in the current study cohort, we did not detect an association of the risk allele of rs3135506 with TG levels in healthy adults or, specifically, in healthy women with lower HDL-C levels. It is plausible that ApoA5, particularly with the SNP rs3135506, could profoundly modulate HDL-C metabolism, even before its direct impact on blood triglyceride levels. One potential hypothesis is that the *APOA5* SNP can mechanistically influence the levels of HDL-C by affecting the maturation of HDL. First, ApoA5 is highly associated with the HDL particles even though the concentration is relatively low compared to other apolipoproteins [63]. Second, it is known that circulating TG levels directly correspond with the TG content in triglyceride-rich lipoproteins (TGRL) [64]. When TG contents rise, ApoA5 moves to TGRL. Conversely, ApoA5 slowly moves back to HDL when TG contents decrease in TGRL after hydrolysis [65]. Lastly, ApoA5 enhances the activity of lecithin-cholesterol acyltransferase (LCAT) that promotes cholesterol efflux [66]. Although all of the above evidence supports that ApoA5 may modulate HDL and cholesterol metabolism, the underlying mechanism by which ApoA5 regulates blood HDL-C concentrations needs to be further elucidated by structural and functional studies.

It is well acknowledged that increased LDL-C level is a risk factor for coronary heart disease [67]. In this study, we found that the risk C allele of rs429358 in *APOE* could significantly increase LDL-C levels in both fasting and postprandial states (Table 3 and Fig. 4). Most importantly, this risk allele also had a negative impact on HDL-C levels in men (Table 4). Moreover, a combination of the risk alleles of rs3135506 and rs429358 significantly augmented the difference in LDL-C levels between the risk allele carriers and non-carriers (Table 5). Studies from others have shown that ApoE acts as a ligand for LDLR [68]. Thus, it has an anti-atherogenic role in reducing the risk of cardiovascular disease by mediating chylomicron and VLDL remnant particle clearance. It has also been shown that the impact of ApoE on LDL-C levels is associated with different isoforms of ApoE, with the impact increasing in the order of ApoE2, ApoE3 and ApoE4, and ApoE4 being detrimental [69]. The C allele of rs429358 is one of the determining elements for the ApoE4 isoform along with the C allele of rs7412. ApoE4 preferentially binds to VLDL while ApoE3 to HDL. This property of binding to VLDL over HDL by ApoE4 is strongly associated with an atherogenic lipoprotein phenotype in ApoE4 carriers. Together, our results suggest that the C allele of rs429358 has a detrimental effect on cholesterol metabolism, especially in men, and hence men carrying this risk allele may be at a higher risk of cardiovascular disease than the non-carriers.

Apart from the finding mentioned above, this study also revealed the association of rs2854116 in *APOC3* with total cholesterol levels and to a lesser extent with LDL-C levels during fasting and postprandially (Table 3 and Fig. 4). Additionally, the current study suggests that this SNP might affect fasting LDL-C levels in women (*P* = 0.07). In a meta-analysis study, the rs2854116 risk allele was positively associated with TG levels while negatively associated with HDL-C levels [70]. Additionally, ApoC3 is primarily associated with cholesterol-rich lipoproteins, such as chylomicrons, VLDL, and LDL [14]. Therefore, as the current study supports, ApoC3 appears to be a key regulator of cholesterol metabolism, in addition to its well documented role in TG metabolism [70]. This theory is also supported by a genetic study performed in a northern French population, which reported a correlation of rs2854116 with elevated LDL-C levels in women [71]. The molecular mechanism of the association remains unclear. The rs2854116 variant is located upstream of the *APOC3* transcription start site. Thus, a plausible explanation would be that the SNP might negatively influence *APOC3* mRNA expression leading to increased rates of conversion of VLDL to LDL particles due to reduced availability of ApoC3 to inhibit lipoprotein lipase-mediated lipolysis [51]. This could subsequently elevate circulating total cholesterol and LDL-C likely induced by a decrease of hepatic uptake of VLDL remnants.

Interestingly, an inverse association of NEFA concentrations in the circulation with the combination of the risk alleles rs2854116 in *APOC3* and rs429358 in *APOE* was found 6-h after lipid challenge (Table 6). Reduced circulating NEFA is correlated with a lower ability to oxidize fat and subsequently liberate NEFA into the plasma from the adipose tissue leading to metabolic deterioration [72, 73]. Furthermore, metabolically healthy obese individuals with high fasting NEFA levels are less likely to develop type 2 diabetes than their insulin-resistant counterparts [74]. Thus, a reduction in 6-h circulating NEFA levels found in the current study may suggest less fat oxidation in subjects carrying the double risk alleles of the SNPs in *APOC3* and *APOE*, which may increase risks of obesity as well as type 2 diabetes in these individuals.

Although strong associations between SNPs and cholesterol profiles were found in this study, there were limitations in the current study that confounded the findings. First, the population size assessed in this study was relatively small, which may hinder significant association of the tested SNPs with TG metabolism as there were dramatic inter-individual differences among the subjects. Second, other phenotypic measurements, such as the sizes of lipoproteins, which contribute to specific functions of their subtypes, and plasma transfer proteins for lipoproteins, including plasma phospholipid-transfer protein (PLTP) and cholesteryl ester transfer protein (CETP), were not measured. Thus, this study could not provide further information on the effects of SNPs on lipoprotein subtypes or interactions with these other important protein modulators of lipoprotein metabolism.

## Conclusions

To the best of our knowledge, the current study is the first to investigate the effect of apolipoprotein allelic variants on blood lipid profiles in healthy adults challenged with a liquid high-fat diet. We identified significant associations of rs3135506 (*APOA5*), rs429358 (*APOE*), and rs2854116 (*APOC3*) with cholesterol metabolism, while the two SNPs in *APOA5* and *APOC3* have been previously recognized as influencers of TG levels. Moreover, we showed that the impact of *APOA5* and *APOE* SNP on HDL-C and LDL-C levels were sex-dependent. Our results suggest that the impact of the SNP variants in the above apolipoproteins might be particularly important for basal cholesterol metabolism but not postprandial cholesterol behavior. These findings provide novel insights into the roles of *APOA5*, *APOE*, and *APOC3* in modulating cholesterol and NEFA metabolism, demonstrating that individuals who carry the non-risk alleles of the tested SNPs of *AOPA5*, *APOE*, and *APOC3* may have reduced genetic risks for cardiovascular disease. The current study also suggests the need for more studies assessing sex-specific risks of cardiovascular disease.

## Supplementary Information

**Additional file 1:** 

**Additional file 2:** **Supplemental Table 1.**  Comparison of the observed SNP frequencies between this study and the 1000 genome project. 

**Additional file 3:** **Supplemental Table 2.**  Distribution of the observed SNP genotype in sex/age/BMI groups. 

**Additional file 4: Supplemental Fig. 1. **Effects of SNPs in *APOA5*, *APOC3*, *APOB*, *APOE*, and *LDLR* on serum triglyceride levels after lipid challenge. Subjects were divided into two genotypic groups separated by sex. A. *APOA5* (*n*=25 and =22 for the CC+CG genotype in men and women, respectively; *n*=142 and =160 for the GG genotype in men and women, respectively). B. *APOC3* (*n*=108 and =122 for the CC+CT genotype in men and women, respectively; *n*=58 and =59 for the TT genotype in men and women, respectively). C. *APOB* (*n*=72 and =82 for the CC+CT genotype in men and women, respectively; *n*=93 and =90 for the TT genotype in men and women, respectively). D. *APOE* (*n*=40 and =56 for the CC+CT genotype in men and women, respectively; *n*=127 and =126 for the TT genotype in men and women, respectively). E. *LDLR* (*n*=31 for the CT+TT genotype in both men and women; *n*=135 and =151 for the CC genotype in men and women, respectively). All data were adjusted for age and BMI and are presented as mean±SE. *,*P*<0.05 between sex groups carrying the same genotype. TG=triglycerides. 

## Data Availability

Requests for published data from the USDA-ARS WHNRC Nutritional Phenotyping Study should be made via an email to the senior WHNRC author on the publication of interest (liping.huang@usda.gov). Requests will be reviewed quarterly by a committee consisting of the study investigators.

## Abbreviations

APOA5: Apolipoprotein A5
APOB: Apolipoprotein B
APOC3: Apolipoprotein C3
APOE: Apolipoprotein 4
HDL-C: High-density lipoprotein-cholesterol
LDL-C: Low-density lipoprotein-cholesterol
TC: Total cholesterol
TG: Triglycerides
TGRL: Triglyceride-rich lipoproteins
NEFA: Non-esterified free fatty acids
CVDs: Cardiovascular diseases
