# Supplementary material for: SNPs in apolipoproteins contribute to sex-dependent differences in blood lipids before and after a high-fat dietary challenge in healthy U.S. adults
Source: BMC Nutr. 2022 Sep 1;8:95. doi: 10.1186/s40795-022-00592-x (PMC9438272; doi:10.1186/s40795-022-00592-x)
Supplement: Supplementary file 2 — Additional file 2: Supplemental Table 1. Comparison of the observed SNP frequencies between this study and the 1000 genome project. [file 40795_2022_592_MOESM2_ESM.docx]

| **Supplemental Table 1** Comparison of the observed SNP frequencies between this study and the 1000 genome project | | | | | | | | | | |
| --- | --- | --- | --- | --- | --- | --- | --- | --- | --- | --- |
|  | *APOA5* | | *APOB* *^a^* | | *APOC3^a^* | | *APOE* | | *LDLR^a^* | |
| SNP ID | rs3135506 | | rs1042034 | | rs2854116 | | rs429358 | | rs2228671 | |
| Allele | C  (%) | G  (%) | G  (%) | A  (%) | C  (%) | T  (%) | C  (%) | T  (%) | T  (%) | C  (%) |
| This study | 6.88 | 93.12 | 26.16 | 73.84 | 42.22 | 57.78 | 14.33 | 85.67 | 9.34 | 90.66 |
| 1000 genome*^b^* | 5.57 | 94.43 | 37.04 | 62.96 | 54.83 | 45.17 | 15.06 | 84.94 | 5.69 | 94.31 |
| *a*, Genotypes were not determined: *APOB*, 2 men (subject IDs 6100 and 6103) and 1 woman (subject ID 8085); *APOC3*, 1 man (subject ID 9049) and 1 woman (subject ID 9067); LDLR, 1 man (subject ID 9024). *b*, Global population. No statistical difference in the SNP frequencies was found between the current cohort and the global population revealed by the 1000 genome project. | | | | | | | | | | |
