# Supplementary material for: SNPs in apolipoproteins contribute to sex-dependent differences in blood lipids before and after a high-fat dietary challenge in healthy U.S. adults
Source: BMC Nutr. 2022 Sep 1;8:95. doi: 10.1186/s40795-022-00592-x (PMC9438272; doi:10.1186/s40795-022-00592-x)
Supplement: Supplementary file 3 — Additional file 3: Supplemental Table 2. Distribution of the observed SNP genotype in sex/age/BMI groups. [file 40795_2022_592_MOESM3_ESM.docx]

| **Supplemental Table 2** Distribution of the observed SNP genotype in sex/age/BMI groups | | | | | | | | | | |
| --- | --- | --- | --- | --- | --- | --- | --- | --- | --- | --- |
| Gene | *APOA5* | | *APOB* *^a^* | | *APOC3^a^* | | *APOE* | | *LDLR^a^* | |
| Genotype | CC+CG  % (n) | GG  % (n) | AG+GG  % (n) | AA  % (n) | CC+CT  % (n) | TT  % (n) | CC+CT  % (n) | TT  % (n) | CT+TT  % (n) | CC  % (n) |
| Men | 15.0 (25) | 85.0 (142) | 43.6 (72) | 56.4 (93) | 65.1 (108) | 34.9 (58) | 24.0 (40) | 76.0 (127) | 18.7 (31) | 81.3 (135) |
| Women | 12.1 (22) | 87.9 (160) | 45.3 (82) | 54.7 (99) | 67.4 (122) | 32.6 (59) | 30.8 (56) | 69.2 (126) | 17.0 (31) | 83.0 (151) |
| Age (y):  18-33  34-49  50-65 | 15.8 (19)  13.8 (16)  10.6 (12) | 84.2 (101)  86.2 (100)  89.4 (101) | 45.8 (55)  46.6 (54)  40.9 (45) | 54.2 (65)  53.4 (62)  59.1 (65) | 65.0 (78)  64.9 (74)  69.0 (78) | 35.0 (42)  35.1 (40)  31.0 (35) | 29.2 (35)  28.4 (33)  24.8 (28) | 70.8 (85)  71.6 (83)  75.2 (85) | 18.3 (22)  22.6 (26)  12.4 (14) | 81.7 (98)  77.4 (89)  87.6 (99) |
| BMI (kg/m^2^)  18.5-24.9  25.0-29.9  30.0-45.0 | 11.1 (15)  14.2 (18)  16.1 (14) | 88.9 (120)  85.8 (109)  83.9 (73) | 49.6 (67)  45.2 (56)  35.6 (31) | 50.4 (68)  54.8 (68)  64.4 (56) | 65.7 (88)  69.3 (88)  62.8 (54) | 34.3 (46)  30.7 (39)  37.2 (32) | 25.2 (34)  29.9 (38)  27.6 (24) | 74.8 (101)  70.1 (89)  72.4 (63) | 17.2 (23)  18.1 (23)  18.4 (16) | 82.8 (111)  81.9 (104)  81.6 (71) |
| *a*, Genotypes were not determined: *APOB* - 2 men and 1 woman in the groups of 50-65 y and BMI 25.0-29.9 kg/m^2^; *APOC3* - 1 man in the groups of 34-49 y and BMI 30-45 kg/m^2^) and 1 woman in the groups of 34-49 y and BMI 18.5-24.9 kg/m^2^); LDLR - 1 man in the groups of 34-49 y and BMI 18.5-24.9 kg/m^2^. The Chi-square test was used to determine the genotypic distribution among categorical variables. No significance was found for the distribution among the categories. | | | | | | | | | | |
