# Supplementary figures and images for: SNPs in apolipoproteins contribute to sex-dependent differences in blood lipids before and after a high-fat dietary challenge in healthy U.S. adults
Source: BMC Nutr. 2022 Sep 1;8:95. doi: 10.1186/s40795-022-00592-x (PMC9438272; doi:10.1186/s40795-022-00592-x)

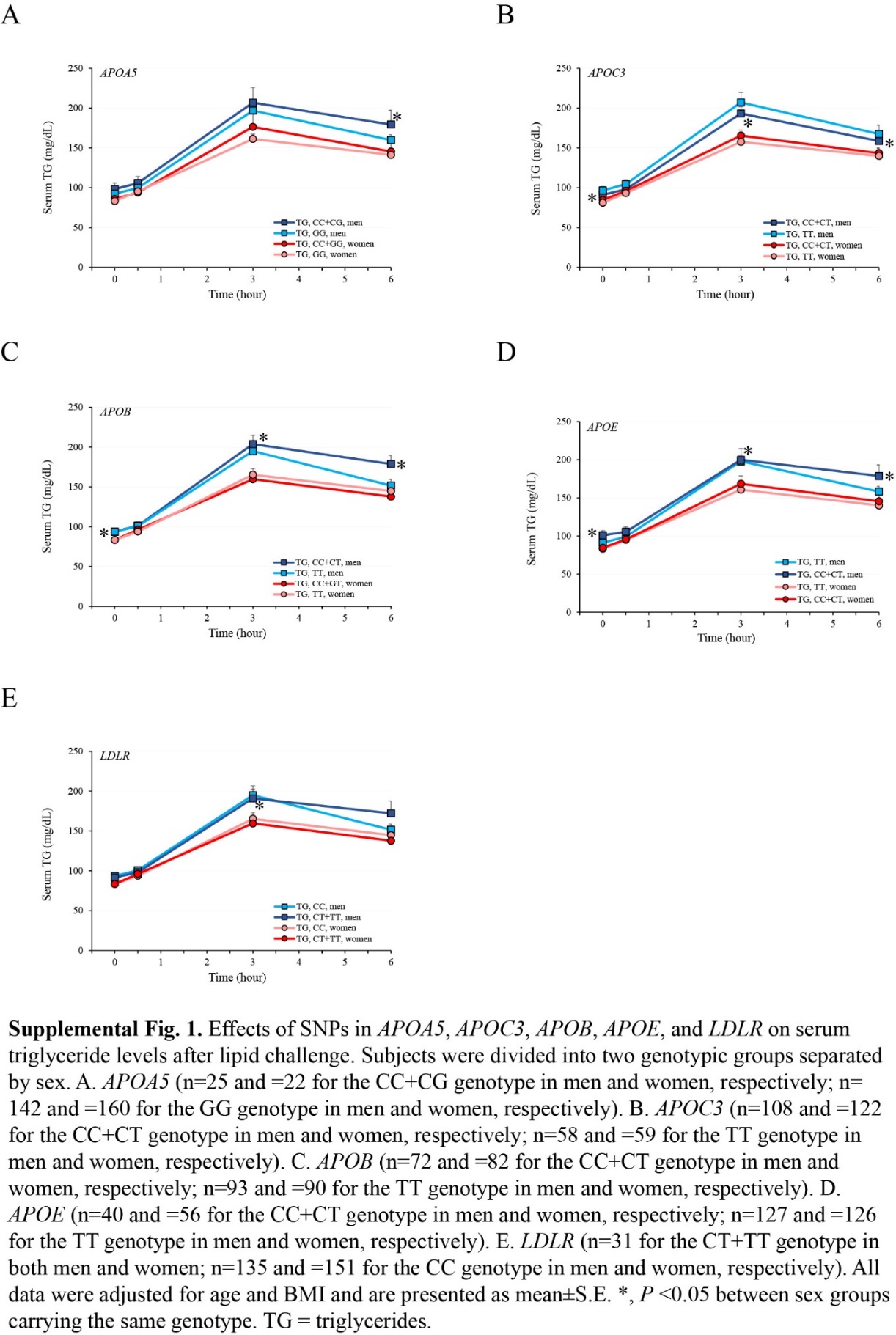

Supplement: Supplementary file 4 — Additional file 4: Supplemental Fig. 1. Effects of SNPs in APOA5, APOC3, APOB, APOE, and LDLR on serum triglyceride levels after lipid challenge. Subjects were divided into two genotypic groups separated by sex. A. APOA5 (n=25 and =22 for the CC+CG genotype in men and women, respectively; n=142 and =160 for the GG genotype in men and women, respectively). B. APOC3 (n=108 and =122 for the CC+CT genotype in men and women, respectively; n=58 and =59 for the TT genotype in men and women, respectively). C. APOB (n=72 and =82 for the CC+CT genotype in men and women, respectively; n=93 and =90 for the TT genotype in men and women, respectively). D. APOE (n=40 and =56 for the CC+CT genotype in men and women, respectively; n=127 and =126 for the TT genotype in men and women, respectively). E. LDLR (n=31 for the CT+TT genotype in both men and women; n=135 and =151 for the CC genotype in men and women, respectively). All data were adjusted for age and BMI and are presented as mean±SE. *,P<0.05 between sex groups carrying the same genotype. TG=triglycerides. [file 40795_2022_592_MOESM4_ESM.docx]
